# Generative haplotype prediction outperforms statistical methods for small variant detection in next-generation sequencing data

**DOI:** 10.1093/bioinformatics/btae565

**Published:** 2024-09-19

**Authors:** Brendan O’Fallon, Ashini Bolia, Jacob Durtschi, Luobin Yang, Eric Fredrickson, Hunter Best

**Affiliations:** Institute for Research and Innovation, ARUP Labs, Salt Lake City, UT 84108, United States; Institute for Clinical and Experimental Pathology, ARUP Labs, Salt Lake City, UT 84108, United States; Institute for Research and Innovation, ARUP Labs, Salt Lake City, UT 84108, United States; Institute for Research and Innovation, ARUP Labs, Salt Lake City, UT 84108, United States; Institute for Clinical and Experimental Pathology, ARUP Labs, Salt Lake City, UT 84108, United States; Institute for Research and Innovation, ARUP Labs, Salt Lake City, UT 84108, United States; Institute for Research and Innovation, ARUP Labs, Salt Lake City, UT 84108, United States; Institute for Research and Innovation, ARUP Labs, Salt Lake City, UT 84108, United States

## Abstract

**Motivation:**

Detection of germline variants in next-generation sequencing data is an essential component of modern genomics analysis. Variant detection tools typically rely on statistical algorithms such as de Bruijn graphs or Hidden Markov models, and are often coupled with heuristic techniques and thresholds to maximize accuracy. Despite significant progress in recent years, current methods still generate thousands of false-positive detections in a typical human whole genome, creating a significant manual review burden.

**Results:**

We introduce a new approach that replaces the handcrafted statistical techniques of previous methods with a single deep generative model. Using a standard transformer-based encoder and double-decoder architecture, our model learns to construct diploid germline haplotypes in a generative fashion identical to modern large language models. We train our model on 37 whole genome sequences from Genome-in-a-Bottle samples, and demonstrate that our method learns to produce accurate haplotypes with correct phase and genotype for all classes of small variants. We compare our method, called Jenever, to FreeBayes, GATK HaplotypeCaller, Clair3, and DeepVariant, and demonstrate that our method has superior overall accuracy compared to other methods. At $F_{1}$-maximizing quality thresholds, our model delivers the highest sensitivity, precision, and the fewest genotyping errors for insertion and deletion variants. For single nucleotide variants, our model demonstrates the highest sensitivity but at somewhat lower precision, and achieves the highest overall $F_{1}$ score among all callers we tested.

**Availability and implementation:**

Jenever is implemented as a python-based command line tool. Source code is available at https://github.com/ARUP-NGS/jenever/

## 1 Introduction

Identifying the DNA sequence variants in a sample is one of the most common and important tasks in modern genomics. Tools that aim to identify variants must overcome several challenges to produce an accurate set of variant calls. For instance, the sequenced reads themselves may contain errors in the form of miscalled bases or PCR-induced repeat length errors, and the tools used to align reads to the reference genome may produce incorrect mappings (e.g. Li 2014, Goldfeder *et al.* 2016). In addition, large or complex sequence variants may be obscured by limitations in the read alignment algorithm, and often require a sophisticated assembly of reads to identify. Reconciling artifacts produced during lab preparation procedures, sequencing, basecalling, and read alignment is a significant technical challenge, and modern variant detection tools employ a variety of statistical and heuristic techniques to achieve high precision and sensitivity (e.g. DePristo *et al.* 2011, Nielsen *et al.* 2011).

All variant discovery tools must address two challenges. First, callers must identify potential alleles in a region. Then, given a set of potential alleles, they must assess the likelihood of each candidate (or pairs of candidates in the diploid case) to determine which should be included in the caller output. Early callers, such as samtools/mpileup (Li *et al.* 2009) and the UnifiedGenotyper tool from the Genome Analysis ToolKit (GATK, DePristo *et al.* 2011) rely on the read aligner to generate candidate alleles, and utilize several *ad hoc* heuristics and thresholds to determine which alleles are most likely to be correct (e.g. Nielsen *et al.* 2011). Later tools (e.g. Kim *et al.* 2018, Poplin *et al.* 2018, Cooke *et al.* 2021) incorporated local re-assembly of reads in each region of interest, resulting in a significant improvement in the sensitivity and precision of variant calls. For instance, the HaplotypeCaller tool identifies candidate haplotypes by constructing de Bruijn graphs from *k*-mers present in the reads, and assesses haplotype likelihoods with a pair Hidden Markov model (pair-HMM). The de Bruijn graph/pair-HMM approach has been adopted by multiple other top-performing tools, including DeepVariant and DRAGEN (Behera *et al.* 2024).

More recent tools have incorporated elements of deep learning into the allele likelihood calculation or allele generation steps. DeepVariant uses a statistical method based on the HaplotypeCaller approach to identify candidates, but adds a convolutional neural net (CNN) to classify variants as true- or false-positive detections. HELLO (Ramachandran *et al.* 2020), designed to work on hybrid short- and long-read datasets, employs a mixture-of-experts approach with separate 1D convolutions across the read and position dimensions of the input. Clair (Luo *et al.* 2019, 2020), uses a novel multitask deep learning approach to predict several properties of a potential variant at a given site, including zygosity and allele length. DAVI (Gupta and Saini 2020) is a unique tool where both primary read mapping and variant detection are performed using neural networks, although the variant detection component was limited to SNPs only and DAVI is unable to reconstruct more complex forms of variation.

Most current variant detection tools that utilize deep learning techniques employ CNNs as the primary architecture (e.g. Gupta and Saini 2020, Luo *et al.* 2020). CNNs have proven successful in computer vision tasks in part due to translational invariance, which helps the model to recognize local features or patterns regardless of their absolute position in an image. In variant detectors, CNNs are often used in an image classification context, such that the primary output is a probability distribution over classes representing, for instance, possible genotypes. However, CNN architectures are less well suited to candidate allele generation, and methods have used statistical approaches to generate candidates, or more complicated multi-task approaches with separate tasks for genotype, allele length, and phasing (e.g. Luo *et al.* 2020).

The transformer architecture (Vaswani *et al.* 2017) offers a more straightforward approach to allele generation than CNNs. Because generative transformer models can create arbitrarily long or complex output sequences, they do not require a separate allele generation step and can be trained end-to-end to produce the desired output. End-to-end training of the full model is promising since the hand-crafted approaches used in prior methods can be replaced by a single, unified model. Recently Baid *et al.* (2023) used a transformer-encoder model to improve the accuracy of PacBio consensus reads, and our group described how aligned next-generation sequencing (NGS) reads can be tokenized in a manner suitable for input to a transformer model (O’Fallon *et al.* 2022), but neither project explored transformer-based decoding. To our knowledge, transformer-based models of haplotype generation have not been explored previously.

Here, we describe a new approach to small variant detection in NGS data that uses a generative, transformer-based model to predict the two germline haplotypes present in the sample. The inputs to our model are the series of bases aligning to individual reference positions, and the outputs are the predicted haplotypes. Each output token of our model is a 4-mer of nucleotide bases, and multiple output tokens are generated to represent full haplotype sequences. This approach does not require any of the bespoke statistical machinery or heuristic thresholds utilized by most modern callers, and instead uses a single deep-learning model to reconstruct complete haplotypes directly from aligned NGS reads.

## 2 Materials and methods

### 2.1 Model architecture and read encoding

Our model consists of a single transformer-based encoder module connected to two transformer decoders, one for each haplotype. The encoder and decoder components are standard transformers with GeLU activations as implemented in PyTorch 2.1 (Vaswani *et al.* 2017, Paszke *et al.* 2019). Input tokens are the collection of bases that align to a given reference position, with some modifications described below. We add an additional fully connected layer prior to the transformer encoders which embeds the encoded basecalls in *d* dimensions, where d=12 for the analyses here. The embedded basecalls are “flattened” along the read dimension, producing an input token with size *dr* where *r* is the number of reads (r=150 for all experiments reported here). *r* defines the maximum number of reads that can be considered in a single window. If fewer than *r* reads are available the input tensor is 0-padded to *r*, and if more than *r* reads are present they are uniformly downsampled to *r*. Instead of the typical 1D positional encoding, we employ a 2D encoding, allowing the model to differentiate across both the token (position) and read dimensions. The 2D encoding implementation follows Wang and Liu (2019).

Input tensors are generated by selecting a 150 bp region from a set of aligned reads in BAM/CRAM format. For each region, all reads overlapping the window were obtained from the corresponding alignment file. Regions containing more than the maximum number of reads (150 for all analyses here) were downsampled to the maximum size. Reads were sorted by the reference coordinate of the first aligned base. For each aligned base in the selected reads ten features are encoded; the first four are the one-hot encoded base call, followed by base quality, two flags indicating if base “consumed” reference base (i.e. was not an insertion) or consumed a sequence read base (was not a deletion), and additional flags indicating sequence read direction, clipping status, and mapping quality. No gap tokens or other special handling was performed for insertions or deletions. In addition to sequenced reads, the first row in each encoded region was the reference sequence. For this special row, we inserted a base quality of 100 for every position and did not set any of the other flags. Resulting tensors had dimension [*g*, *r*, 10], where *g* indexes genomic positions, *r* indexes reads, and 10 represents the number of features. Positions in the input not corresponding to an aligned base were all set to zero.

**Figure 1.**
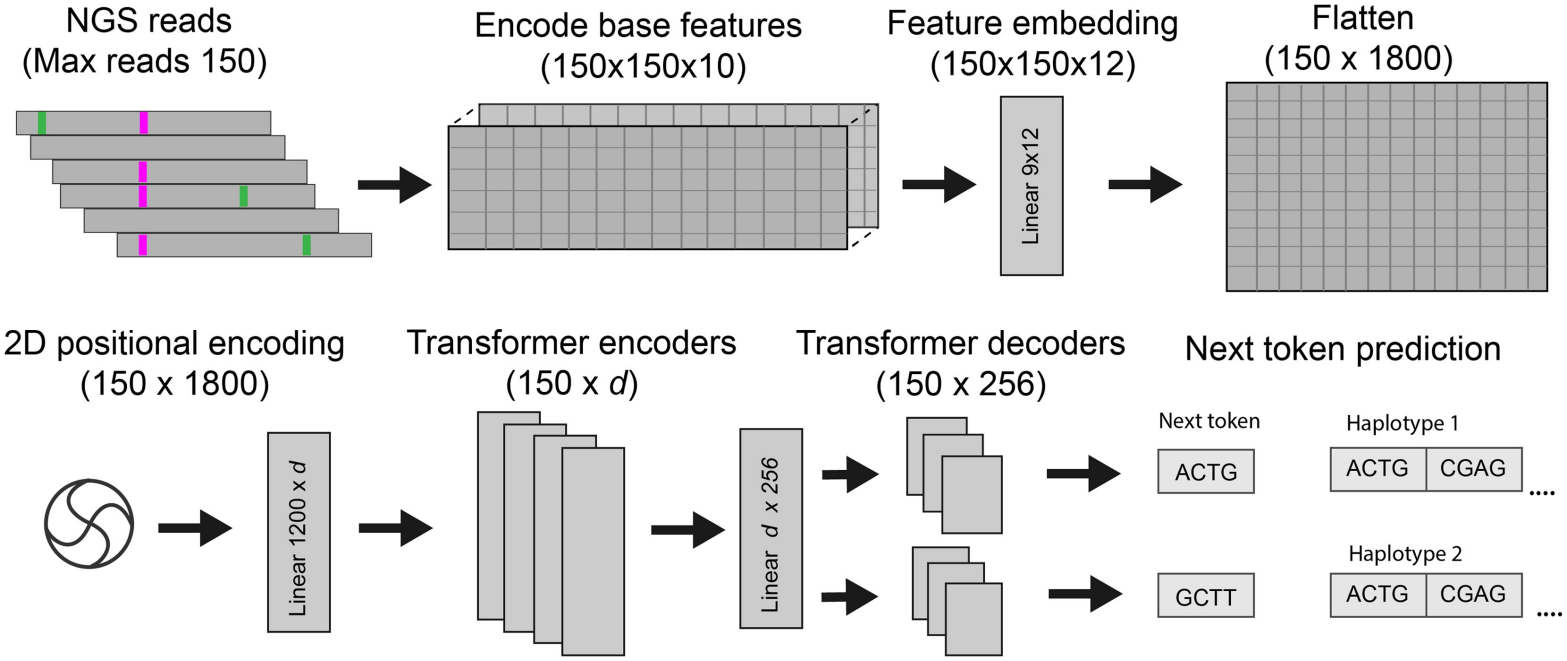
Model architecture for Jenever.

Our model uses two parallel transformer decoder blocks, each of which produces output tokens representing a single haplotype. Output tokens are *k*-mers of length 4, and the decoders do not share parameters. As there are four possible bases at each position both decoders have model dimension $4^{4}=256$. Because the encoder and decoders do not necessarily have equal dimensions we convert encoder logits to dimension 256 with a single linear layer. A standard 1D positional encoding is applied to the encoder logits prior to decoding (see Fig. 1).

### 2.2 Training data and procedure

Training data were obtained from 37 whole genome sequences (WGS) from seven Genome-in-a-Bottle cell lines. Ten of the samples were prepared with the Illumina TruSeq PCR-free kit, while the others were prepared with the Illumina Nextera DNA Flex kit, which involves several PCR cycles. All samples were sequenced on an Illumina NovaSeq 6000 instrument in 2 × 150 mode, to an approximate read depth of 50 (range 24.7–72.3). After conversion to fastq, the sequenced reads were aligned to human reference genome GRCh37.p13 with the GEM-mapper (v3, Marco-Sola *et al.* 2012) and were sorted and converted to CRAM format with samtools version 1.9 (Li *et al.* 2009). No additional refinements, such as duplicate read marking, base-quality score recalibration, or indel realignment were performed.

To select regions to include for training data, we developed a scheme to sample regions in a biased manner, prioritizing regions containing variants and, especially, regions with multiple or complex variants. Regions of the reference genome overlapping the high-confidence regions from Genome-in-a-Bottle were subdivided into 150 bp windows, and these regions labeled according to the presence of variants. Separate labels were generated for regions containing a Single Nucleotide Variant (SNV), deletion, or insertion, as well as regions containing multiple insertion-deletions, or those containing variants intersecting low-complexity or poor mappability regions. We additionally included “true negative” regions where no known variant was present. In regions containing more than 150 overlapping reads, the reads were repeatedly downsampled to generate multiple training regions. For all analyses described here, we obtain training data only from the human autosomes 1 to 20, and hold out chromosomes 21 and 22 for model evaluation. Approximately 1.48M regions were obtained from each of the 37 samples, generating a full training dataset of 54.8M regions encompassing 8.2B input tokens.

Target haplotype sequences were produced by obtaining truth variants from the Genome-in-a-Bottle VCF files (version 4.2.1, Wagner *et al.* 2022) for each sample and inserting the variants into the reference sequence. Two sequences were generated for each region, one representing each haplotype. In regions where phasing of the variants was ambiguous, the reads in the sequenced sample were examined to determine phase status. Briefly, all possible genotypes (pairs of haplotypes) were generated and reads were aligned via Smith-Waterman to the possible haplotypes, and the highest-scoring genotypes selected as the most likely phasing. This phasing procedure was only attempted for variants <100 bp apart, otherwise the region was discarded.

Models were trained using the AdamW optimizer with β weight decay parameters set to 0.9 and 0.99, as implemented in PyTorch 2.1 (Paszke *et al.* 2019). We used a learning rate schedule with a linear warm-up from a value near 0 to a maximum of $5\times 10^{-5}$, followed by cosine decay to a minimum of $10^{-5}$, and a batch size of 512 regions. For our best-performing model, we performed additional fine-tuning rounds on data with increased representation of poor mappability and segmentally duplicated regions as defined in the Genome-in-a-Bottle stratification files (version 4.2, Wagner *et al.* 2022). These fine-tuning runs were performed with a learning rate schedule as above, but with a maximum rate of $10^{-5}$ and minimum of $10^{-6}$. Training was performed on 1–2 Nvidia A6000 (Ampere generation) GPUs.

Haplotypes have no intrinsic order, but the model produces two haplotypes that must be matched to the corresponding target haplotypes correctly. If the model produces haplotypes $\left(A_{P},B_{P}\right)$ and the target haplotypes are $\left(B_{T},A_{T}\right)$, we want to compute the loss and corresponding gradients for $L(A_{P},A_{T})+L(B_{P},B_{T})$, where *L* is the loss function. For instance, the model should not be penalized for producing haplotypes with an SNV on haplotype *A* when the targets have the same SNV on haplotype *B*. To ameliorate this, we compute the loss for each haplotype configuration and backpropogate gradients only for the configuration that produces the lowest loss.

### 2.3 Variant detection

**Figure 2.**
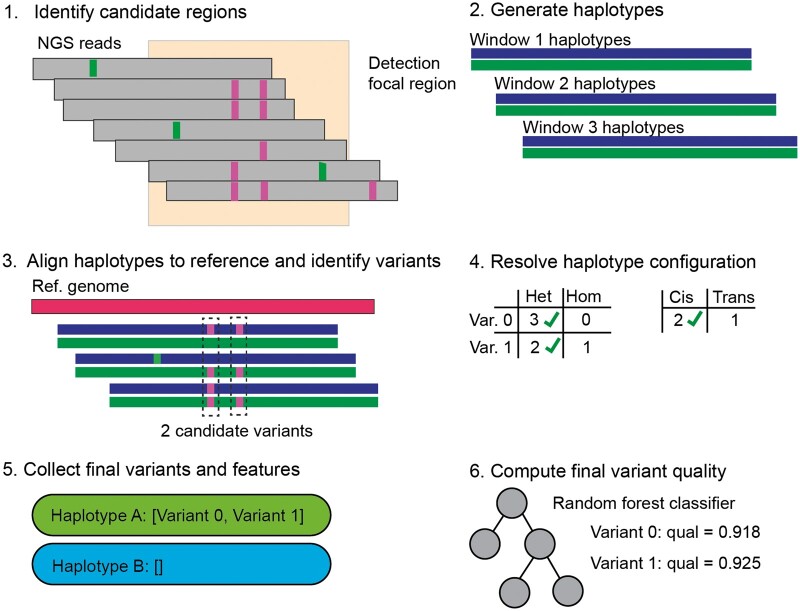
Variant calling procedure in six steps, see text for explanation.

The variant detection procedure consists of six steps, shown in Fig. 2. Given an alignment file in BAM or CRAM format, we first identify regions where a potential variant might exist (Fig. 2, step 1). Any genomic position in which at least three reads contain a base that differs from the reference or an indel is flagged as potentially containing a variant. All regions are padded by four basepairs in both directions, and regions closer than 100 bp are merged into a single region.

For a single region containing suspected variants, we perform multiple overlapping forward passes of the model with step size *k*, where k=25 for the results reported here, beginning 100 bases upstream of the potential variant (Fig. 2, step 2). We use the standard greedy decoding procedure to generate haplotypes, selecting the single 4-mer with the highest predicted score on each iteration until the predicted sequence spans the entire candidate region or until 37 4-mers (148 bases) have been generated. Each haplotype is aligned via Smith-Waterman to the reference sequence, and any mismatching positions are converted to variant calls (Fig. 2, step 3). After variants are collected across multiple windows, results are merged using an *ad hoc* method that attempts to minimize the number of conflicting calls between windows using a simple majority-rules algorithm (Fig. 2, step 4). For instance, if a variant was found to be heterozygous in two windows and homozygous in one window, the output variant is predicted to be heterozygous. For each variant, we record the number of windows in which each variant was detected, the number of variants in cis and trans, the probability associated with the *k*-mer containing the variant bases, and the position of the variant within each window (Fig. 2, step 5).

### 2.4 Variant quality calculation

The above variant detection procedure has two shortcomings. First, if every potential variant is emitted, then precision is poor. Second, the model does not innately produce well-calibrated variant quality scores. To address these shortcomings, we introduce a *post hoc* random forest classifier that we train to discriminate true- and false-positive variant calls. The resulting score is used as a final variant quality score suitable for tuning the tradeoff between sensitivity, precision, and the false-discovery rate (FDR; Fig. 2, step 6). Higher cutoffs correspond to lower sensitivity but improved FDR, which may be desirable in applications that require high specificity. Similar approaches for calibrating NGS variant data have been discussed previously (e.g. Li *et al.* 2019, Köster *et al.* 2020), although our solution differs in that it utilizes features unique to our method, such as token logits and window offsets. The random forest classifier was trained by calling variants on selected regions from autosomes 1 to 2 totaling approximately 90 MB. True- and false-positive variant calls were identified with the vcfeval tool (Cleary *et al.* 2015). We used the scikit-learn implementation of the random forest with 100 trees and maximum tree depth of 25. Features are listed in Supplementary Table S1.

## 3 Results

### 3.1 Model size and training data experiments

**Figure 3.**
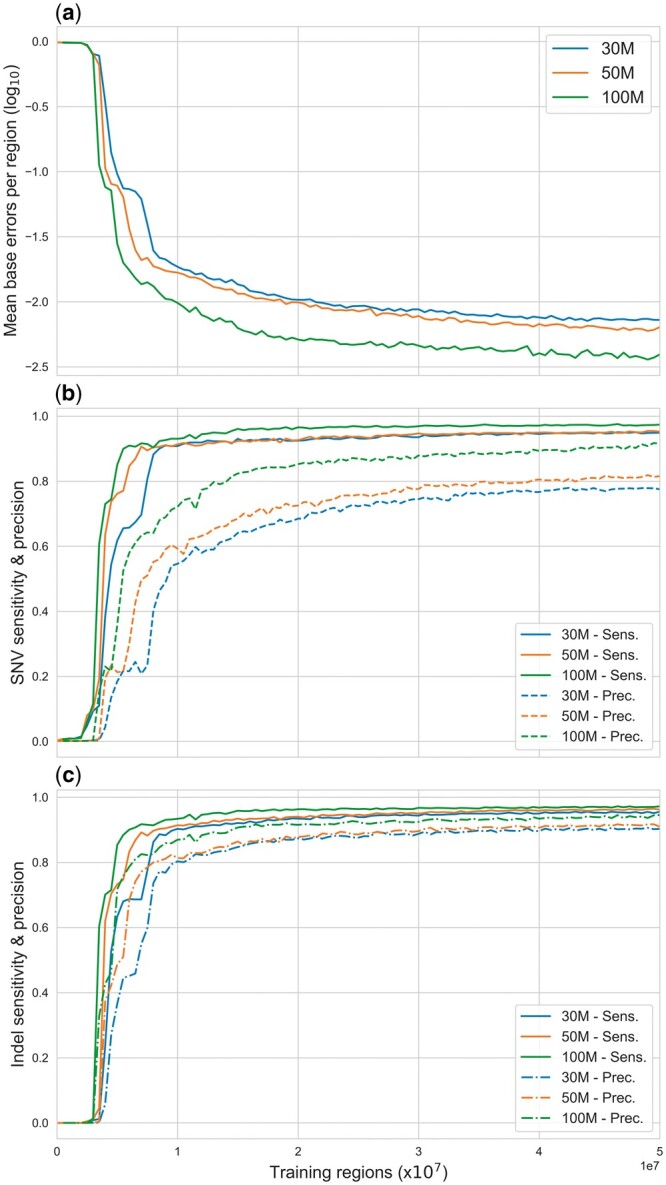
Mean base prediction error rate (a), sensitivity and precision curves for SNVs (b), and indels (c) for the chromosome 21 and 22 validation regions during the first epoch of training for the 30M, 50M, and 100M models. Base error rate is the mean fraction of incorrectly predicted bases in a region. Data are collected for individual validation regions prior to the multiple window calling procedure and variant quality calculations.

We examined performance for three different model sizes (Table 1). Overall model accuracy, sensitivity, and precision increased with model size and over the course of training (Fig. 3), with most of the gains accruing during the first $3\times 10^{7}$ training regions processed. Precision increased more slowly than sensitivity during training, with raw SNV precision reaching only 80% for the 30M and 50M models after one epoch. Training for additional epochs improved performance somewhat, with SNV precision reaching approximately 98% for the 100M model after three epochs. Note that these training evaluation statistics do not reflect the full variant detection procedure outlined in Section 2.3, and are derived from a single window without variant quality calculation or filtering. Sensitivity was higher overall and showed less variability across model sizes, although the 100M model consistently demonstrated a slightly higher SNV accuracy than the smaller models.

**Table 1.** Model parameter combinations used for the three model sizes investigated.

Model name	Encoder layers	Encoder heads	Embedding dim.	Decoder layers	Decoder heads
30M	6	8	800	4	4
50M	8	8	960	8	4
100M	10	8	1280	10	10

### 3.2 Variant detection accuracy

To characterize variant detection accuracy, we called variants on validation regions from chromosomes 21 and 22 for all 37 samples following the procedure described in section 2.3, and compared results to DeepVariant (v1.3.0), GATK HaplotypeCaller (v. 4.1.4.1), FreeBayes 1.3.6 (Garrison and Marth 2012), and Clair3 (Luo *et al.* 2020). The regions include most of chromosomes 21 and 22, although we omit the p-arm of chromosome 21 which is almost entirely masked on GRCh37, in total comprising about 67.8 MB. For this comparison, we used our largest model (100M), after training for 3.1 epochs (approximately 336 h of training time on two Nvidia A6000 GPUs) and hand-selecting the model checkpoint associated with the lowest validation loss. We selected filtering criteria for each caller by reviewing ROC curves produced by vcfeval (Cleary *et al.* 2015) and selecting values close to the $F_{1}$ maximizing value across samples. Jenever calls were filtered at quality 10 (phred-scaled), HaplotypeCaller at 50, Clair3 at 0, and DeepVariant at 3. Sensitivity, precision, and related calculations were computed with the *hap*.*py* tool (Krusche *et al.* 2019) using Genome-in-a-Bottle (v4.2.1) as the benchmark variant set.

**Figure 4.**
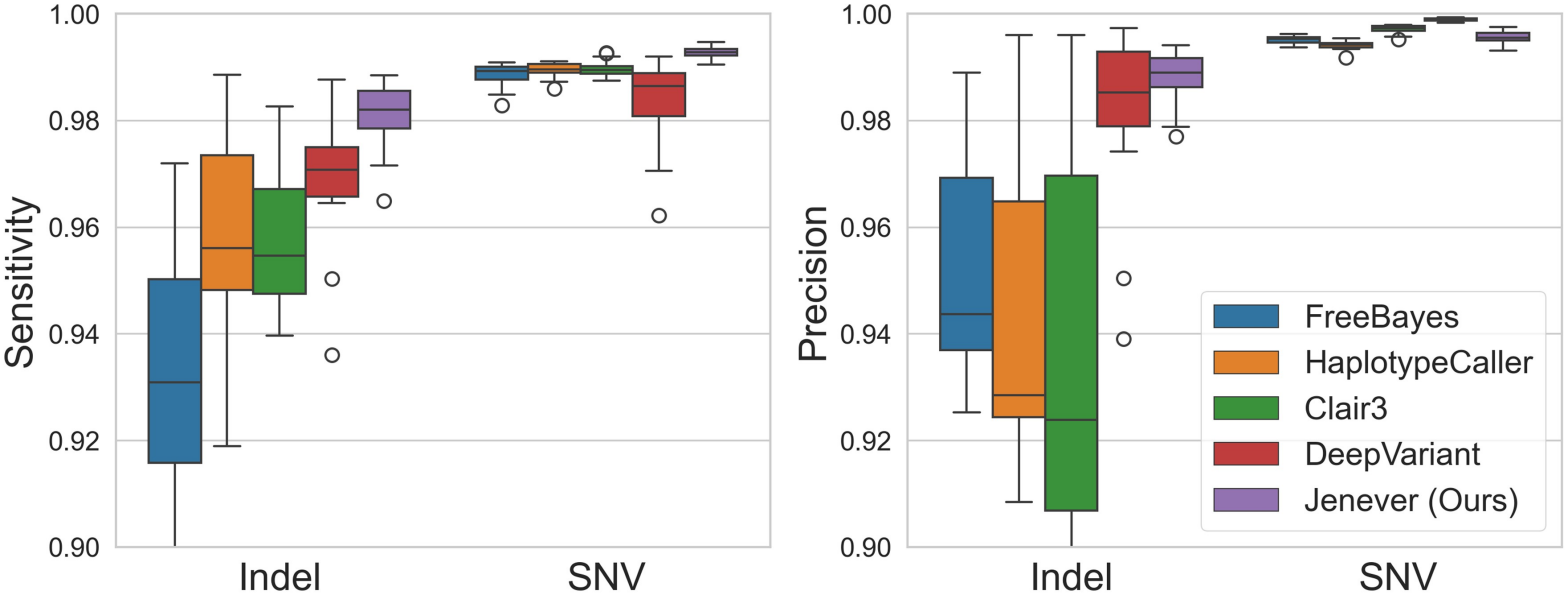
Mean per-sample sensitivity (a) and precision (b) for indels and SNVs in validation regions as computed by *hap*.*py* across variant callers. Error bars denote 95% confidence intervals.

**Figure 5.**
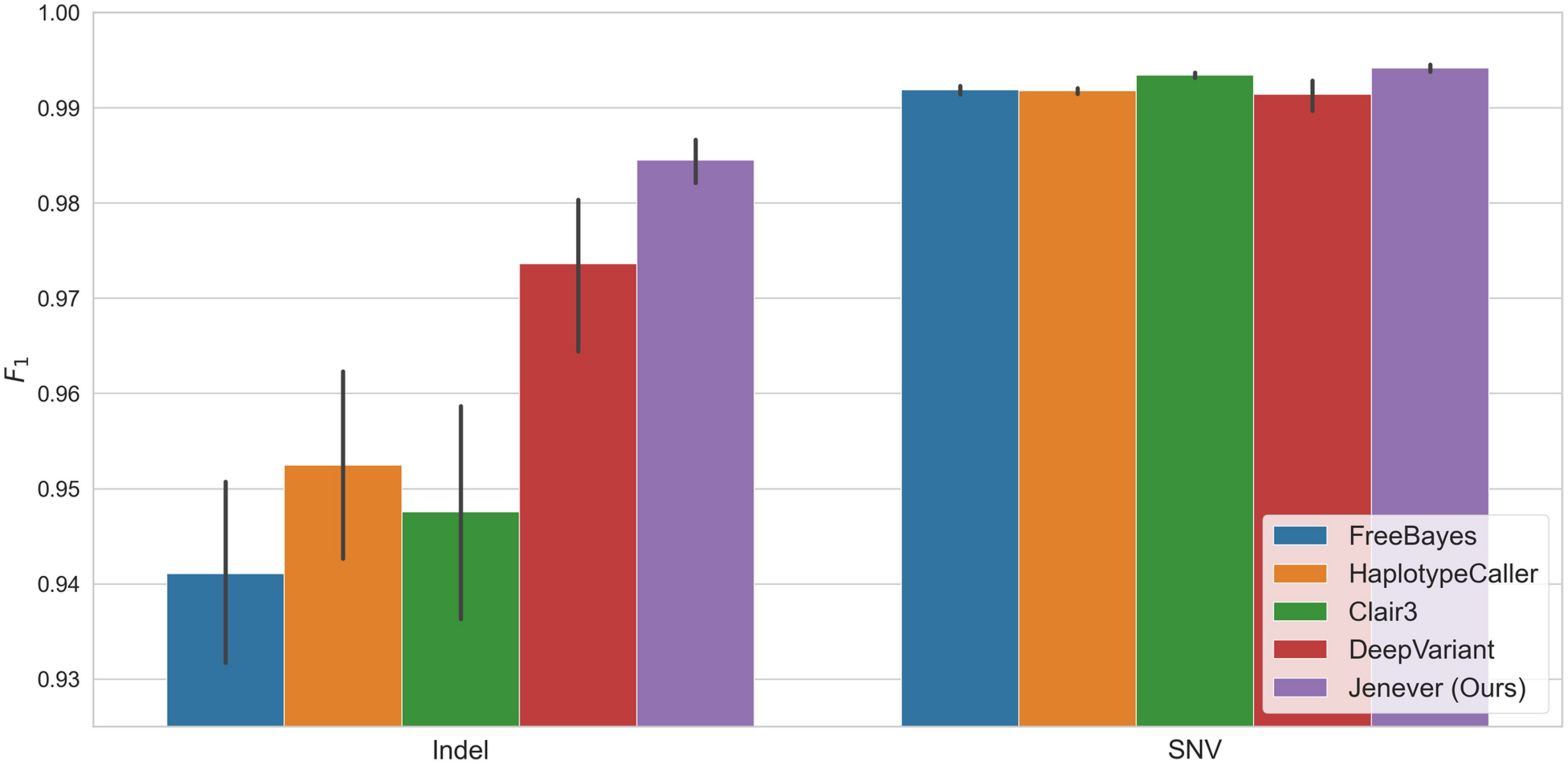
Mean per-sample $F_{1}$ scores for variants in validation regions, as computed by *hap*.*py* for all variant callers examined. Error bars denote 95% confidence intervals.

Jenever achieved the highest sensitivity and precision for insertion/deletion (indel) calls across all callers we tested, with a mean sensitivity of 98.09% and precision of 98.81% (Fig. 4). DeepVariant was the next closest in performance, with a sensitivity of 96.49% and a precision of 98.26%. For single nucleotide variants (SNVs), our model had the highest sensitivity (99.27%) of all callers, but was outperformed by both DeepVariant and Clair3 in precision, with DeepVariant achieving the highest overall precision of 99.89% and Jenever yielding 99.56%. Overall, our model had the highest $F_{1}$ score for both indels (98.45%) and SNVs (99.42%; Fig. 5), surpassing the nearest competitor, DeepVariant, by more than a full percentage point (1.08%) for indels and 0.3% for SNVs.

**Figure 6.**
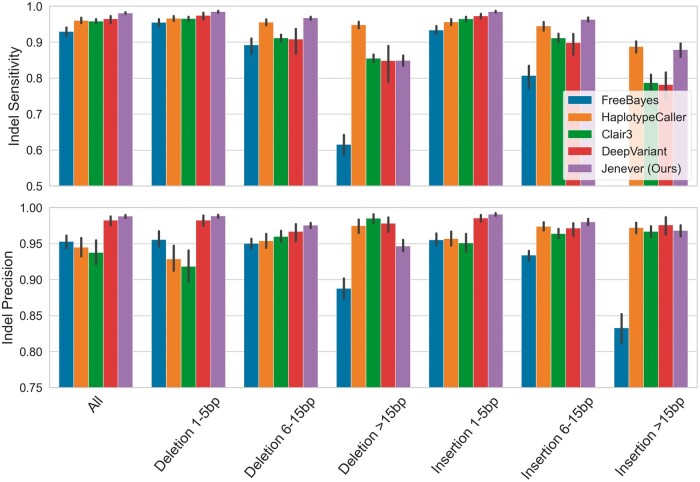
Sensitivity and precision for insertion and deletion variants binned by size. Error bars denote 95% confidence intervals.

Jenever showed especially high accuracy for small (1–5 bp) to medium size (6–15 bp) insertions and deletions, and both insertion and deletion variants had similar sensitivity and precision. For large deletions (>15 bp) Jenever had somewhat lower precision than other callers, but similar sensitivity, while Jenever demonstrated high sensitivity and average precision for insertions > 15 bp (Fig. 6).

### 3.3 Genotype and phasing accuracy

A key feature of our model is the use of two parallel transformer decoder blocks to generate haplotypes. The decoder blocks do not communicate, leaving the encoder block as the only mechanism to “decide” which haplotype each decoder must generate. To assess the accuracy of this system we examine both the genotype predictions of individual calls and the phase predictions for pairs of nearby calls by comparing them to Genome-in-a-Bottle reference data.

**Figure 7.**
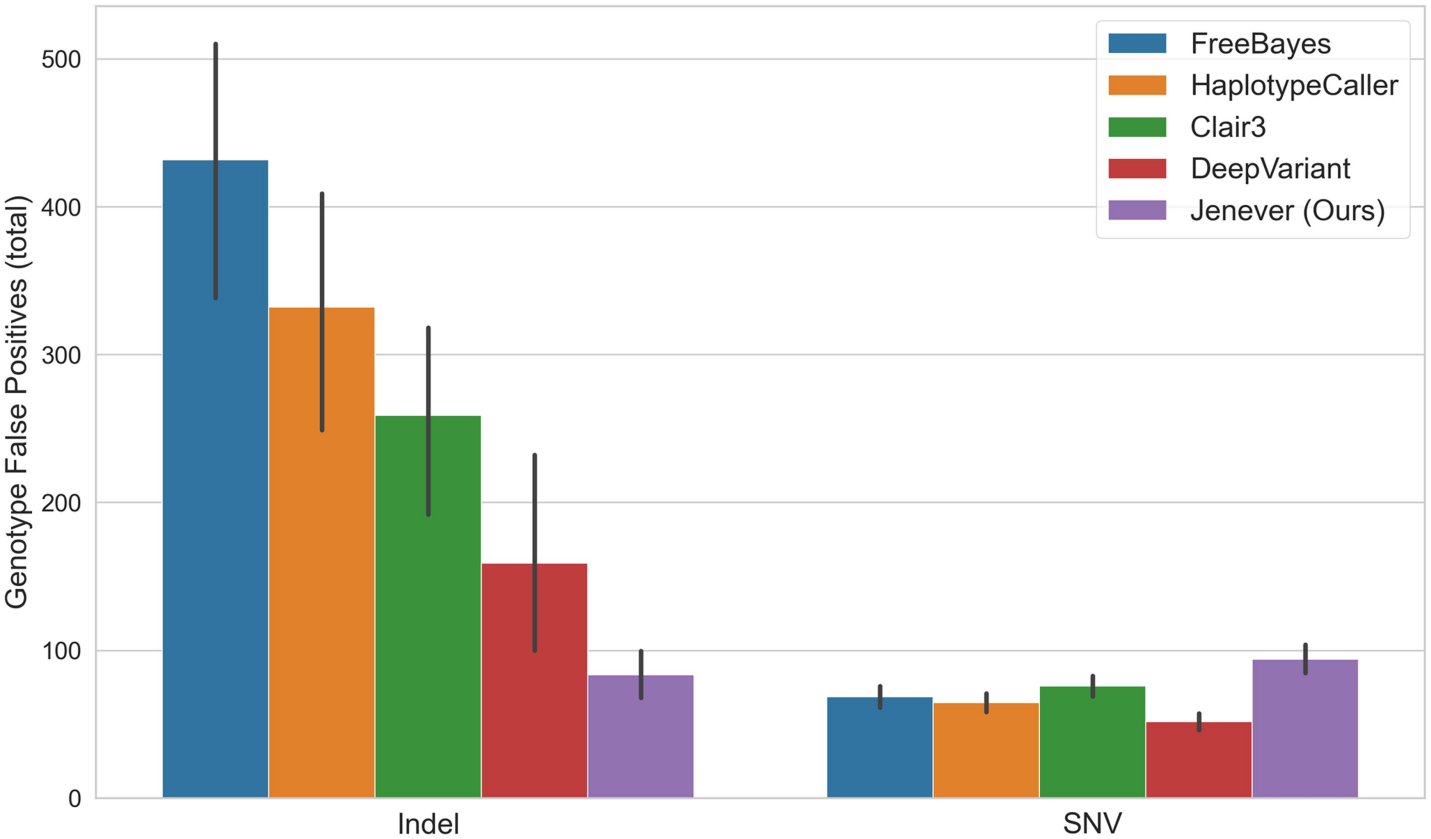
Mean number of incorrect genotype calls for indels and SNVs in validation regions for all callers examined.

We examined genotype accuracy by tallying the number of variants in validation regions where the callers produced the correct allele and position, but the incorrect genotype, as computed by *hap*.*py*. Jenever produced the fewest incorrect genotype calls for indel variants (mean 83.4 per sample), but the most incorrect genotypes for SNVs (mean 93.8) (Fig. 7).

By explicitly modeling haplotypes, our method provides a form of read-backed phasing. We investigate the accuracy of our phase predictions by comparing them to data from Genome-in-a-Bottle reference sample HG002, the only reference sample for which phasing data are provided. Because the reference used pedigree information to phase the variants we cannot match most of the predictions, and instead we assess how often the phase predictions from our model match those from the reference sample. Specifically, we find groups of phased variants in our predictions, and for each adjacent pair of variants in the same phase group, we compare to the reference sample. Occurrences in which our phase predictions matched or mismatched those from the reference were tallied, and cases where no phase information was provided from the reference, or variant genotype was found to conflict with the reference, were discarded. For this analysis, we discarded variants with a phred-scaled quality score of <10, in a manner identical to the previous section.

Phase accuracy was high overall and decreased with distance between variants (Table 2). Over 95% of variant pairs have phase predictions that matched the reference sample when variants were within 50 bp, but accuracy drops to just over 80% for variants that are >100 bp apart. Anecdotally, most incorrect predictions appear in pairs involving indels in low complexity sequence.

**Table 2.** Accuracy of phase predictions compared to reference sample HG002 for pairs of variants in chromosome 21–22 validation regions.

Distance (bp)	Correct phase	Incorrect phase	Phase precision (%)
1–24	12 726	315	97.6
25–49	9737	315	96.9
50–100	13 303	802	94.3
>100	197	40	83.2

### 3.4 Runtime performance

**Figure 8.**
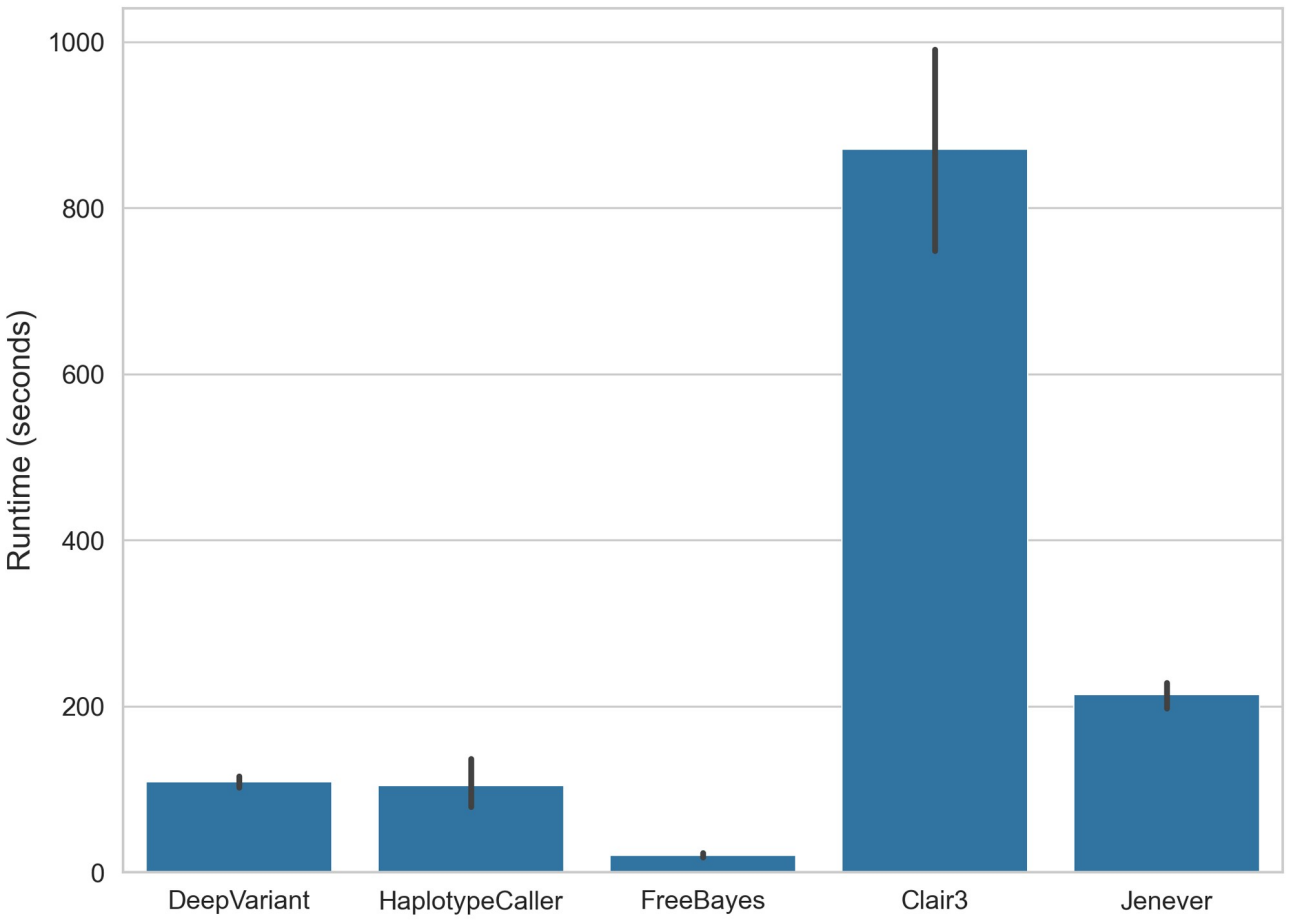
Total number of seconds required to call variants over a 1 MB region of chromosome 21.

We assessed the runtime performance of Jenever and the other callers by selecting a 1 MB region from chromosome 21 and recording the total execution time of the calling command. Processes were given access to a single Nvidia A6000 (Ampere generation) GPU and 24 CPU cores, with resources managed by the slurm HPC cluster manager. FreeBayes was the fastest caller by a substantial margin, completing the calling procedure in approximately 20 s for each sample (Fig. 8). Both HaplotypeCaller and DeepVariant had similar performance and completed in approximately 100 s. Jenever was substantially slower and took on average 214 s, while Clair3 was the slowest caller and took 870 s to finish processing each sample.

## 4 Discussion

We describe a new approach to detecting sequence variants in short-read NGS data that utilizes a deep generative model of haplotypes. Our model takes short NGS reads aligned to the reference genome as input, and learns to predict the two germline haplotypes present in the sample. The model architecture is very similar to that of modern large language models (LLMs), but we replace the input “prompt” with aligned NGS reads, and instead of generating words, we generate nucleotide *k*-mers representing haplotypes. The advantage of this approach is that the model fully encompasses both the candidate allele generation and evaluation components, and thus can be optimized directly to produce accurate haplotypes. In contrast to other common variant detection tools, our approach does not require any handcrafted statistical methods, bespoke algorithms, or finely-tuned thresholds.

Our model is significantly more accurate than existing small variant detectors, especially for insertion and deletion variants. At quality thresholds chosen to maximize the $F_{1}$ statistic, our model delivers a mean sensitivity more than 1.5% higher than the next closest model (98.0%, compared to 96.5% for DeepVariant) at improved precision (98.8%, compared to 98.2% for DeepVariant). Additionally, our model makes half as many genotyping errors for indels (variants where the correct allele was detected but with the wrong genotype) as the next closest competitor (Fig. 7). For single nucleotide variants (SNVs), results are more mixed. Both DeepVariant and Clair3 offer higher precision at $F_{1}$ maximizing quality thresholds, but at the cost of significantly reduced sensitivity. Our model produces more false-positive calls and more genotyping errors, but detects many additional true positive calls that are missed by other callers, leading to a higher $F_{1}$ accuracy overall. Jenever’s mean precision for SNVs was 99.5% while DeepVariant delivered an impressive 99.9%; however, DeepVariant’s sensitivity was only 98.4%, while our model found 99.3% of real variants.

We hypothesize that the performance difference between indels and SNVs in our model may be due to the attention mechanism used in the transformer architecture. Both the self- and cross-attention mechanisms in the transformer architecture were originally designed for language modeling (Vaswani *et al.* 2017) and rely on computing the interaction between tokens, where tokens represent information across genomics positions. Intuitively, this seems well-suited to inferring indel variants, where the model must utilize information from multiple nearby tokens to produce the correct output. In contrast, SNV detection typically requires utilizing information from a single genomic position. Manually reviewing some of the incorrect SNV calls suggests that Jenever struggles in differentiating true and false calls at lower allele frequencies, a task where multi-token attention is less useful. In an earlier work, we demonstrated that a similar model used the number of nearby reference mismatches to inform SNV detection for a given site (O’Fallon *et al.* 2022), suggesting that the model can, in some cases, employ information from the local context to improve SNV detection, but this mechanism appears less accurate than convolutional approaches used in other modern models.

While our implementation is very accurate, it is slower than most other tools. Generation of each output token (4-mer) requires a separate pass through both haplotype decoders, each of which is a deep neural net with tens of millions of parameters, and each candidate region requires multiple passes of the model for high prediction accuracy. Despite this, numerous avenues exist for improving runtime performance, including model quantization, pruning, distillation, multi-query attention (Shazeer 2019), and many others. In general, improving transformer inference speeds is a topic of intense interest in both the academic and business communities, with recent significant advances in both software (e.g. Dao *et al.* 2022) and hardware. We plan to explore methods for improving runtime performance in future work.

By framing NGS variant detection as a transformer-based deep generative model, we are able to leverage the many recent advances in transformer training and inference optimizations. For instance, improvements in positional embeddings (e.g. Shaw *et al.* 2018, Su *et al.* 2024), attention mechanisms (e.g. Choromanski *et al.* 2020, Roy *et al.* 2021), ensembling methods (e.g. Riquelme *et al.* 2021) or training methodologies (e.g. Izmailov *et al.* 2018), although originally designed for language or vision transformers, are likely to provide benefits for our model as well. By combining these and other advances, numerous opportunities exist for delivering the next generation of variant callers.

## Supplementary Material

btae565_Supplementary_Data

## Data Availability

All source code and training scripts are available via git at https://github.com/ARUP-NGS/jenever.
