# Supplementary material for: Generative haplotype prediction outperforms statistical methods for small variant detection in next-generation sequencing data
Source: Bioinformatics. 2024 Sep 19;40(11):btae565. doi: 10.1093/bioinformatics/btae565 (PMC11549014; doi:10.1093/bioinformatics/btae565)
Supplement: btae565_Supplementary_Data [file btae565_supplementary_data.docx]

**Supplementary Information**

| Feature | Description |
| --- | --- |
| Mean probability | Mean probability (exponentiated logit) associated with first token containing variant across windows |
| Ref length | Length of reference allele |
| Alt length | Length of longest alternate allele |
| Min probability | Minimum probability (exponentiated logit) of first token containing variant across windows |
| Max probability | Maximum probability (exponentiated logit) of first token containing variant across windows |
| Window var count | Total number of variants in all windows |
| Cis count | Total number of variants on same haplotype as this variant |
| Trans count | Total number of variants on opposite haplotype as this variant |
| Step count | Total number of windows in which this variant was called |
| Window count | Total number of windows overlapping this variant position |
| Min window offset | Minimum distance in basepairs from variant position to start of calling window |
| Max window offset | Maximum distance basepairs from variant position to start of calling window |
| Depth | Total number of reads overlapping this variant position |
| Genotype | 1 if variant is heterozygous, 0 otherwise |
| Variant allele frequency | Fraction of reads containing this variant allele |
| Read support | Total number of reads containing this variant allele |

Supplementary Table 1: Features used to train the variant quality score model
